# The Book World of Medicine and Science

**Published:** 1898-09-10

**Authors:** 


### THE BOOK WORLD OF MEDICINE AND SCIENCE.

GUIDE TO THE CLINICAL EXAMINATION AND TREATMENT OF SICK CHILDREN. By JOHN THOMSON, M.D., F.R.C.P.Ed. Pp. 336, post 8vo. Fifty-two illustrations. (Edinburgh: William F. Clay. 1898. Price 9s.)

This book, as the author states, is an expansion of some clinical lectures delivered at the Royal Hospital for Sick Children, Edinburgh, and it has suffered from the circumstances of its origin. Like most books expanded from lectures, it is too incomplete to serve as a text-book, and, at the same time, its defects of form prevent its being a quite satisfactory clinical manual. The specific fevers, for example, are barely referred to, and, while whole chapters are devoted to rickets, feeding, gastric disorders, and the like, congenital syphilis is only incidentally mentioned in the directions for examination of the mouth, skin, &c. The chapters on physical examination are in many ways inadequate, and the physical signs of pulmonary disorders only sketchily indicated. In the account of the skin no mention is made of those curious complaints œdema and sclerema neonatorum. Yet, with all its faults of arrangement, the book is in many ways an exceedingly good one. The chapter on "Growth and Development" is sympathetically written and of great clinical value; the sections on hygiene, therapeutics, and food disorders are excellent. Perhaps Soxhlet's steriliser and the practice of milk sterilisation generally are too leniently dealt with, and the value of Pasteurisation hardly sufficiently insisted on. The account given of "Mental Deficiency" is far better than what is usually written on this subject. In its present form this book will doubtless be appreciated by many, and in future editions, with some rearrangement and amplification, it will probably take high rank. An appendix with some therapeutic formulæ is given, and scattered through the book are many photographs.

APPLIED BACTERIOLOGY. By T. H. PEARMAIN and C. G. MOOR, M.A. Second edition. Pp. 464, 8vo. (London: Baillière, Tindall, and Cox. 1898. Price 12s. 6d. net.)

This, the second edition of Messrs. Pearmain and Moor's text-book, has, the authors state, undergone thorough revision and partial expansion. Yet, after carefully re-reading it, we are bound to notice the persistence of not a few faults. In the first place an undue amount of space is devoted, at the expense of crowding out much that is purely technical, to matters not within the province of the bacteriologist at all. This is the more serious when we find that the nosological matter is at best only secondhand. Again, in the account of the bacteriology of syphilis, we have the authors' opinion of the C.D. Acts; and of the pages devoted to influenza two-thirds are given up to symptomatology. The account of the Tricophyton Tonsurans completely ignores the work of Sabouraud. Under the heading of "Cholera" we can find no account of Pfeiffer's reaction or Metchnikoff's serum, while the great question of "unity" is merely touched on. It would be well if, in another edition, the superfluous nosological matter were deleted, and if attention were confined to the strictly bacteriological aspect of the subject.

DISEASES AND REMEDIES: A CONCISE SURVEY OF THE MOST MODERN METHODS OF MEDICINE. Written expressly for the Drug Trade by Physicians and Pharmacists. (London: The *Chemist and Druggist* Office. 1898.)

This little book is one of a class with which we have but little patience. The attempt to teach the art of medicine in 225 small pages would be absurd, even if the maxims given were intended only for use in emergencies when far removed from professional help. This handbook, however, appears to be intended for the use of chemists and druggists as a guide to counter-prescribing. It is maintained in the preface that there is a form of counter-prescribing which is legitimate, necessary, and inevitable, and the chief interest of the volume before us seems to lie in the light that it throws upon the amount of knowledge which is required for the exercise of this gentle art.

RESPONSIBLE OR IRRESPONSIBLE? CRIMINAL OR MENTALLY DISEASED? A PLEA FOR THE UNJUSTLY CONVICTED AND ON THE CAUSE OF CRIME. By HENRY SMITH, M.D. (Jena). (London: Watts and Co., 17, Johnson's Court, Fleet Street. 1898. Price 1s.)

Dr. Smith's little book deals with a subject of such immense importance that we could wish he had been a little less emotional in his treatment of it. That occasionally a person may be treated as a criminal who is really a madman with an irresistible kind of impulse to a certain kind of crime is true, but we doubt if these cases are as frequent as the author would make out. He, indeed, seems to think that all crime is evidence of disease, which, indeed, it may be, and yet require punishment. For a moral disease is not the same thing as a physical or intellectual one, and for it the ordinary treatment of imprisonment, with or without hard labour, may be as good as any that can be devised. We think that any well-marked case of insanity always gets full consideration, but it is certain, though the author does not seem to take it into account, that if a slight eccentricity of conduct was sure to secure an acquittal, the ordinary criminal would not be slow to affect the symptoms of insanity on the smallest provocation. To admit insanity as an extenuating circumstance in cases in which it can be proved is one thing, to assume that every criminal is *ipso facto* a lunatic, as we fancy from his little book Dr. Smith inclines to do, is another, and, we think, a very dangerous thing, too, for it is opposed to human nature and to fact.

ASEPSIS AND ANTISEPSIS IN ABDOMINAL SURGERY AND GYNÆCOLOGY. By H. MACNAUGHTON JONES, M.D., F.R.C.S. Pp. 63. Illustrated. (London: Baillière, Tindall, and Cox. 1898. Price 3s. net.)

In this little book Dr. MacNaughton Jones given an account of the arrangements of his private operating-room, and of the methods—aseptic and antiseptic—that he employs. Dr. Jones' practice does not appear to differ in these matters to any notable extent from that of other present-day surgeons. Hence there is little that is novel in these pages. The book is freely illustrated by engravings of the ordinary appliances of the operating theatre, and not a few photographs. These latter, which include some pleasing pictures of surgeon, assistants, and nurses ready for operation, appear a little superfluous. They convey no instruction to the professional reader, and we cannot suppose they are intended to attract the general public. A brief account of the methods of certain continental surgeons is given in the last chapter.

### BOOKS RECEIVED.

BAILLIÈRE, TINDALL, AND COX.

